# Characterization of the Virulence and Yield Impact of *Fusarium* Species on Canola (*Brassica napus*)

**DOI:** 10.3390/plants12173020

**Published:** 2023-08-22

**Authors:** Haitian Yu, Kan-Fa Chang, Sheau-Fang Hwang, Stephen E. Strelkov

**Affiliations:** 1 Department of Agricultural, Food and Nutritional Science, University of Alberta, Edmonton, AB T6G 2P5, Canada; haitian7@ualberta.ca (H.Y.); kanfa@ualberta.ca (K.-F.C.); 2 Institute of Food Crops, Yunnan Academy of Agricultural Science, Kunming 650205, China

**Keywords:** agronomic traits, *Brassica napus*, *Fusarium* species, interspecific interactions, yield loss model

## Abstract

Multiple species of *Fusarium* can contribute to the development of root rot in canola (*Brassica napus*), making disease management difficult. We conducted field and greenhouse experiments to investigate the impacts of *Fusarium avenaceum* and *Fusarium proliferatum*, and the interaction between *Fusarium oxysporum* and *F. proliferatum* on root rot severity and canola yields. Inoculation with any of the three *Fusarium* spp. resulted in significant disease severity and reduced seedling emergence compared with non-inoculated controls, leading to yield reductions of up to 35%. Notably, there was a strong correlation (*r* = 0.93) between root rot severity at the seedling stage and at maturity. Regression analysis indicated a linear decline in seedling emergence with increasing disease severity. Furthermore, disease severity at maturity adversely affected the pod number per plant and the seed weight per plant, with both parameters ultimately approaching zero at a severity of 4.0 on a 0–4 scale. Co-inoculation with *F. oxysporum* and *F. proliferatum* induced more severe root rot than inoculation with each species on its own, suggesting synergistic interactions between these fungi. Knowledge of these interactions and the relative virulence of *Fusarium* spp. will contribute to the improved management of root rot in canola.

## 1. Introduction

Canola (*Brassica napus* L.) is an important crop on the Canadian prairies, contributing an average of $10.0 billion annually in farm cash receipts between 2017 and 2021 [1]. The production of this crop, however, can be constrained by root rot, a soilborne disease caused by a complex of pathogens, including *Fusarium* spp., *Rhizoctonia solani*, and *Pythium* spp. [2]. Root rot is commonly associated with seed decay, damping off, and the decomposition of infected root tissues, resulting in substantial yield losses, particularly under conditions favorable for disease development [3,4]. Partial or nearly complete yield losses have been reported because of root rot in canola [5,6,7,8]. In western Canada, *R*. *solani* is one of the primary pathogens responsible for root rot and damping off [7]. However, *Fusarium* species, including *F*. *oxysporum* and *F*. *avenaceum*, also play crucial roles in the root rot complex [2,9,10,11]. In recent years, *F*. *proliferatum* has been reported with increasing frequency as a cause of root rot, crown rot, yellowing, and wilting in various vegetable, fruit, and field crops [12,13,14,15]. Several *Fusarium* spp., including *F*. *proliferatum*, were the most frequently isolated fungi in diseased canola root and stem samples collected from central and northern Alberta, Canada, in 2021 [9]. Inoculating canola with these *F*. *proliferatum* isolates resulted in significant reductions in seedling emergence and plant height, confirming the pathogenicity of this fungus on canola [9]. The wide range of hosts susceptible to *Fusarium* spp. [12,16], coupled with their ability to survive as saprophytes and produce long-lived resting structures (chlamydospores), makes these fungi challenging to manage [17]. Furthermore, a recent study [18] suggests that the projected increase in temperatures due to climate change may increase the occurrence and severity of root rot and related diseases in the coming decades.

While the cultivation of resistant crops is widely recognized as a cost-effective and environmentally friendly strategy for disease management [19], the availability of resistance to Fusarium root rot appears to be restricted [20]. Additionally, the effectiveness of crop rotation and other cultural management strategies can be limited for this disease [21], primarily due to the broad host range of *Fusarium* spp., which limits options for non-susceptible crops to include in the rotations. Fungicide seed treatments have been widely applied to manage soilborne pathogens [22,23], but their efficacy against different *Fusarium* spp. varies [24,25]. While one study investigated the impacts of seed size, fungicidal seed treatment, seeding depth, and seeding date on canola seedling blight caused by *F*. *avenaceum* [3], the capacities of different *Fusarium* spp. to cause root rot in canola, and their effects on yields, have not been evaluated. Moreover, both *F*. *proliferatum* and *F*. *oxysporum* have been identified as capable of invading the xylem, causing similar symptoms including wilting and xylem discoloration [26,27]. These shared patterns in disease progression imply similarities in the interactions between these *Fusarium* species and the host plant. This study aimed to enhance understanding of the potential impacts of *F*. *proliferatum*, *F*. *avenaceum*, and *F*. *oxysporum* on canola production, and had three specific objectives: (1) assessing the abilities of different *Fusarium* spp. to cause root rot; (2) evaluating the effects of *Fusarium* spp. on seedling emergence and yield; and (3) investigating the interrelationship between *F*. *proliferatum* and *F*. *oxysporum* in disease development and growth reduction in canola.

## 2. Results

### 2.1. Temperature and Rainfall

Precipitation and temperature at the St. Albert Research Station differed between 2021 and 2022, particularly in June, July, and September (Table S1 and Figure S1). The precipitation totals in June and July of 2022 surpassed those of 2021, with values of 129.3 mm and 43.3 mm, respectively, compared with 41.6 mm and 28.7 mm in the previous year.

Monthly mean temperatures, as well as the maximum and minimum temperatures, were higher in August and September of 2022 in comparison with those of 2021. Nonetheless, very high temperatures (>30 °C) occurred earlier in 2021, specifically during the final week of June. The daily maximum temperatures during that period ranged from 30.7 °C to 35.9 °C. In contrast, a maximum temperature of >30 °C did not occur until July 28 in 2022, while the highest recorded temperature (33.7 °C) was in early September. No spell of very high temperatures lasted more than three consecutive days during the 2022 growing season. 

### 2.2. Effects of F. proliferatum and F. avenaceum under Greenhouse Conditions

**Figure 1:**
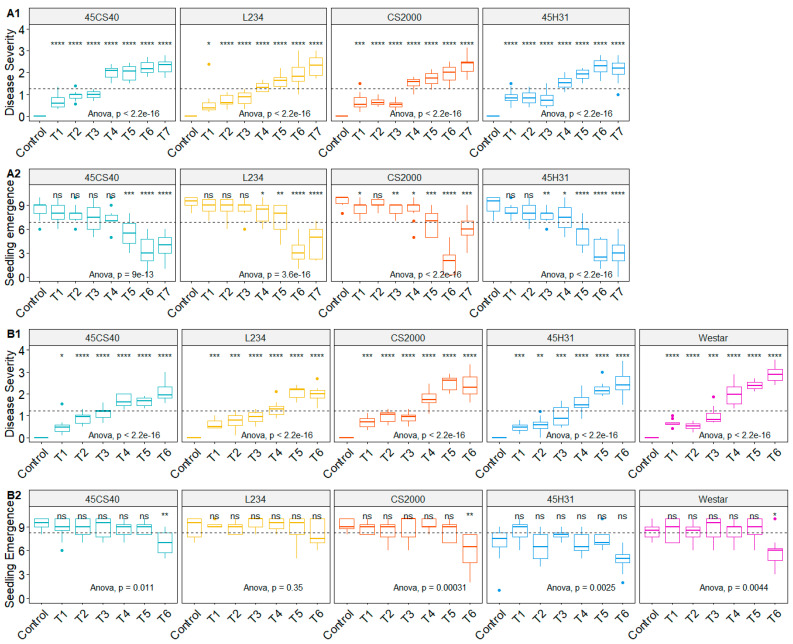
Comparison of the effect of inoculation with *Fusarium proliferatum* on root rot severity (**A1**) and seedling emergence (**A2**) in the canola cultivars ‘45CS40’, ‘L234’, ‘CS2000’, and ‘45H31’, and *Fusarium avenaceum* on root rot severity (**B1**) and seedling emergence (**B2**) in the canola cultivars ‘45CS40’, ‘L234’, ‘CS2000’, ‘45H31’and ‘Westar’, under greenhouse conditions. (**A1**,**A2**): Control, non-inoculated control of each cultivar; T1, 1:2000 (*v*/*v*) ratio of *Fusarium proliferatum* (*Fp*) to potting mix; T2, 1:1000 (*v*/*v*) ratio of *Fp* to potting mix; T3, 1:400 (*v*/*v*) ratio of *Fp* to potting mix (*v*/*v*); T4, 1:200 (*v*/*v*) ratio of *Fp* to potting mix; T5, 1:133 (*v*/*v*) ratio of *Fp* to potting mix (*v*/*v*); T6, 1:100 (*v*/*v*) ratio of *Fp* to potting mix; T7, 1:67 (*v*/*v*) ratio of *Fp* to potting mix. (**B1**,**B2**): Control, non-inoculated control of each cultivar; T1, 1:49 (*v*/*v*) ratio of *Fusarium avenaceum* (*Fa*) to potting mix (*v*/*v*); T2, 1:24 (*v*/*v*) ratio of *Fa* to potting mix (*v*/*v*); T3, 3:47 (*v*/*v*) ratio of *Fa* to potting mix; T4, 2:23 (*v*/*v*) ratio of *Fa* to potting mix; T5, 3:22 (*v*/*v*) ratio of *Fa* to potting mix; T6, 3:17 (*v*/*v*) ratio of *Fa* to potting mix. ns, no significant difference between the treatment and corresponding control based on a *t*-test; *, significant difference at *p* ≤ 0.05; **, significant difference at *p* ≤ 0.01; ***, significant difference at *p* ≤ 0.001; and ****, significant difference at *p* ≤ 0.0001. The dashed lines represent the overall mean for each parameter.

In nearly all cases, canola seedling emergence was reduced significantly following inoculation with *F. proliferatum* or *F. avenaceum* (Figure 1). Particularly large reductions in emergence were observed under moderate to high inoculum concentrations. The only exception was for ‘L234’ after inoculation with *F. avenaceum*, where a significant reduction in emergence was not detected. 

As expected, non-inoculated treatments did not develop any symptoms of root rot (disease severity = 0). In contrast, treatment with any concentration of *F. proliferatum* (Figure 1(A1,A2)) or *F. avenaceum* (Figure 1(B1,B2)) inoculum resulted in a significant increase in disease. Root rot generally became more severe with increasing inoculum concentration, with average ratings of 2.3 and 2.1 in ‘45CS40’, 2.3 and 2.0 in ‘L234’, 2.4 and 2.4 in ‘CS2000’, and 2.1 and 2.5 in ‘45H31’, following inoculation with the highest rates of *F. proliferatum* and *F. avenaceum*, respectively. ‘Westar’, which was treated only with *F. avenaceum*, developed a root rot severity rating of 2.9 at the highest inoculum concentration.

### 2.3. Effects of F. proliferatum and F. avenaceum under Field Conditions

In the two field experiments, both the year and the interactions between year and treatment had a significant effect on most parameters tested, including yield and emergence. Additionally, notable site effects were observed in 2022 for certain parameters (emergence after inoculation with *F. avenaceum*), although the interactions between site and other factors were largely non-significant. Consequently, for subsequent analysis, the data sets from the two years were examined separately, without considering the site effect.

In 2021, inoculation with *F. proliferatum* resulted in a reduction in yield, while simultaneously increasing root rot severity compared with the non-inoculated control (Table 1). Both cultivars exhibited a significant increase in disease severity with increasing inoculum concentration, while emergence decreased from the control to the moderate inoculum concentration. The yield of cultivar ‘CS2000’ experienced a significant reduction (as high as 29.80%) under high inoculum concentration. Cultivar ‘45H31’, on the other hand, incurred yield losses ranging from 6.96% to 10.00%. Likewise, in 2022, inoculation with *F. proliferatum* had detrimental effects on yield, emergence, and disease severity. The root rot severities in both cultivars were significantly higher than those of their respective controls under all inoculum levels, and increased with escalating inoculum concentration. The seedling emergence of ‘45H31’ declined across all inoculum levels, while the emergence of ‘CS2000’ was reduced at moderate to high inoculum concentrations. Inoculation had a significant effect on the yield of ‘45H31’, with yield losses of 12.76%, 13.01%, and 25.77% under low, moderate, and high inoculum concentrations, respectively. Likewise, for ‘CS2000’, all treatments also led to a significant decrease in yield, ranging from 18.16% to 31.37%. 

In 2021, both cultivars exhibited reduced yield and emergence, as well as increased root rot severity, following inoculation with *F. avenaceum* (Table 2). Yield losses ranged from 9.04% to 19.68% for ‘45H31’ and from 7.21% to 17.79% for ‘CS2000’. Similarly, in 2022, there were consistent decreases in both yield and emergence for both cultivars, while disease severities exhibited significant increases with increasing *F. avenaceum* inoculum level (Table 2). In that year, yield losses ranged from 21.07% to 33.66% for ‘45H31’ and from 22.53% to 30.38% for ‘CS2000’. 

In general, for both *Fusarium* species, the performance outcomes of the two cultivars with respect to emergence, root rot severity, and yield were not significantly different over the two years of the experiment.

### 2.4. Co-Inoculation with F. proliferatum and F. oxysporum under Greenhouse Conditions

**Figure 2:**
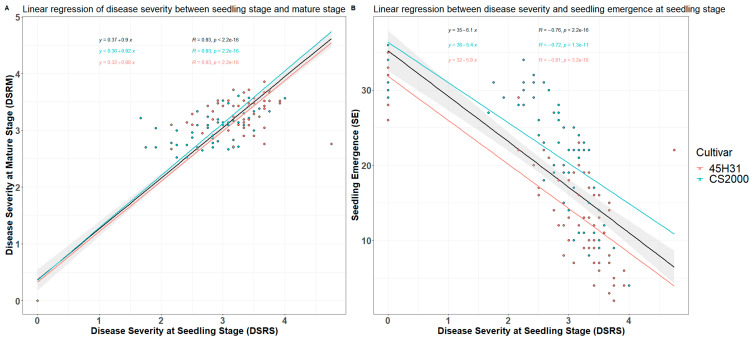
Linear regression between average canola root rot disease severity at the seedling stage and at maturity (**A**), and between average disease severity at the seedling stage and seedling emergence (**B**) for all treatments under greenhouse conditions. Black line, linear regression based on the combined data for the cultivars ‘45H31’ and ‘CS2000’; pink line, linear regression for ‘45H31’; blue line, linear regression for ‘CS2000’. DSRS, root rot disease severity (0–4 scale) at the seedling (21 days after seeding) stage; DSRM, root rot disease severity (0–4 scale) at maturity; SE, seedling emergence (number of plants) per box.

The ANOVA conducted on the two repeats of this experiment confirmed that variances in emergence, disease severity at the seedling stage, and disease severity at maturity were homogeneous. As a result, the data from the two repeats were combined. However, for yield parameters (pod number and seed weight per experimental unit) with significantly distinct variances between the two repeats (*p* < 0.01), separate analyses were conducted. Linear regression analysis indicated a highly significant correlation (*p* < 0.001) between root rot severity at the seedling stage and at maturity. This correlation was strong for both the combined data for the two cultivars and for the data for each individual cultivar (*r* = 0.93). Additionally, disease severity at the seedling stage had a significant effect (*p* < 0.001) on emergence, with correlation coefficients of approximately −0.80 (Figure 2). The cultivar ‘CS2000’ showed greater seedling emergence and lower disease severity across all treatments relative to ‘45H31’.

Nearly all of the inoculated treatments had significantly lower emergence and more severe disease relative to the controls. Root rot severity varied between 2.99 and 3.60 for cultivar ‘45H31’, and between 2.56 and 3.39 for ‘CS2000’ (Table 3). The 75 mL Fp: 75 mL Fo: 5000 mL soilless mix treatment showed the lowest emergence, but the highest disease severity, for both cultivars. No significant difference was found between the 22.5 mL Fp: 75 mL Fo: 5000 mL soilless mix treatment and the 75 mL Fp: 22.5 mL Fo: 5000 mL soilless mix treatment, which had equivalent total levels of grain inoculum applied.

Treatment with 37.5 mL Fp: 37.5 mL Fo: 5000 mL soilless mix resulted in reductions in nearly all parameters, including seedling emergence, seed weight, and pod number. Additionally, significant differences were observed in terms of emergence and root rot severity at maturity when comparing this treatment to inoculation with 75 mL Fp: 0 mL Fo: 5000 mL soilless mix or 0 mL Fp: 75 mL Fo: 5000 mL soilless mix. In comparison to treatments with 75 mL Fp: 0 mL Fo: 5000 mL soilless mix or 0 mL Fp: 75 mL Fo: 5000 mL soilless mix, the treatment with 22.5 mL Fp: 22.5 mL Fo: 5000 mL soilless mix (which combined both Fusarium spp.) had a nearly identical impact on most parameters, despite containing less grain inoculum overall. Seed weight and pod number per box decreased significantly in the two repeated trials under all inoculated treatments, with the decline becoming greater as the amount of grain inoculum increased (Table 4); reductions exceeding 50% were observed at the higher inoculation rates. 

**Figure 3:**
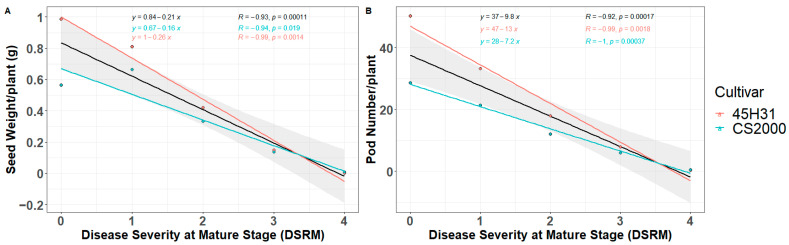
Linear regression between canola yield parameters (seed weight per plant (**A**) and pod number per plant (**B**)) and root rot disease severity at maturity. Black line, linear regression based on the combined data for the cultivars ‘45H31’ and ‘CS2000’; pink line, linear regression for ‘45H31’; blue line, linear regression for ‘CS2000’. DSRM, root rot disease severity (0–4 scale) at maturity; Seed Weight/plant, the average seed weight for all plants at each DSRM level (0–4); Pod Number/plant, the average pod number for all plants at each DSRM level (0–4).

Significant negative correlations (*p* < 0.05) were found between disease severity at maturity and both seed weight and pod number. These correlations held true for each cultivar, as well as when analyzing the combined data of the two cultivars. Similarly, a linear decrease in seed weight and pod number per plant was observed with increasing disease severity at maturity. As disease severity approached 4.0, the reduction in these parameters reached as high as 100% (Figure 3). These findings indicate a strong association between disease severity at maturity and adverse effects on seed weight and pod number.

### 2.5. Effects of Co-Inoculation with F. proliferatum and F. oxysporum under Field Conditions

In the co-inoculation field experiments, variance was homogeneous for yield, disease severity, and emergence between the two sites (*p* > 0.05). For the other parameters, the *p*-values ranged from 0.01 to 0.05, suggesting no significant interaction between sites, treatments, or cultivars. As a result, the data from both sites were combined for the subsequent analysis.

All of the inoculated treatments experienced a detrimental effect on disease severity, emergence, and yield for both cultivars when compared with their respective non-inoculated controls (Table 5). The disease severity in all of the inoculated treatments was significantly greater than in the non-inoculated controls. No significant differences were observed, however, among the various inoculated treatments. Disease severity in the controls remained consistently low (<0.40). In contrast, for cultivar ‘45H31’, disease severity ranged from 1.58 to 1.89 in the inoculated treatments, while for ‘CS2000’, it ranged from 1.60 to 1.86. Similarly, the seedling emergence of both cultivars was reduced by all of the inoculations. Treatment with a mixture of 50 mL Fp: 50 mL Fo: 0 mL sand resulted in the lowest emergence rate. In addition, treatment with a combination of 25 mL Fp: 25 mL Fo: 70 mL sand showed significantly lower emergence compared to the treatment with 50 mL Fp: 0 mL Fo: 50 mL sand or treatment with 0 mL Fp: 50 mL Fo: 50 mL sand. However, no significant differences were observed between treatment with 50 mL Fp: 0 mL Fo: 50 mL sand and treatment with 0 mL Fp: 50 mL Fo: 50 mL sand, or between treatment with 15 mL Fp: 50 mL Fo: 35 mL sand and treatment with 50 mL Fp: 15 mL Fo: 35 mL sand.

Almost all of the treatments significantly reduced the yields of both cultivars. Yield losses for ‘45H31’ ranged from 10.30% in the treatment with 15 mL Fp: 15 mL Fo: 70 mL sand to 34.67% in the treatment with 50 mL Fp: 50 mL Fo: 0 mL sand. No significant differences in yield were observed among treatments receiving the same amount of total grain inoculum, irrespective of whether they were applied individually or in combination. In a similar fashion, for cultivar ‘CS2000’, yield losses ranged from 12.24% (in the treatment with 50 mL Fp: 0 mL Fo: 50 mL sand) to 30.16% (in the treatment with 50 mL Fp: 50 mL Fo: 0 mL sand). However, a significant difference was observed when comparing the treatment with 25 mL Fp: 25 mL Fo: 50 mL sand to the treatment with 50 mL Fp: 0 mL Fo: 50 mL sand, with the former showing a notably lower yield. In contrast, no significant differences were found between the treatment with 50 mL Fp: 0 mL Fo: 50 mL sand and the treatment with 0 mL Fp: 50 mL Fo: 50 mL sand, nor between the treatment with 15 mL Fp: 50 mL Fo: 35 mL sand and the treatment with 50 mL Fp: 15 mL Fo: 35 mL sand. No significant differences were detected among the canola cultivars for yield, seedling emergence, or disease severity for any of the treatments.

## 3. Discussion

The management of root rot in canola can be difficult, particularly given the involvement of multiple *Fusarium* species in disease development. The minimal inoculum densities required to induce disease, whether under natural conditions or in vitro, varied depending on the virulence of the strains [2,28,29,30,31]. In our study, we employed pre-identified densities as distinct treatments to accurately simulate the disease-conducive inoculum conditions in nature. In this study, the application of *F*. *avenaceum*, *F*. *proliferatum,* or *F*. *oxysporum* at the seedling stage resulted in severe disease and reduced emergence. These findings are consistent with previous research by Chen et al. [10], who observed that *Fusarium* spp. collected from soil and diseased canola plants in central Alberta caused significant seedling blight and root rot, with *F*. *avenaceum* in particular found to be highly aggressive on canola. Moreover, in the current study, disease severity increased with higher inoculum concentrations of both *F*. *proliferatum* and *F*. *avenaceum*, leading to decreases in emergence. These results align with similar studies on canola, in which *F*. *avenaceum* was found to reduce seedling emergence [2], as well as studies on bean, in which *Fusarium solani* f. sp. *phaseoli* caused severe root rot [32], and on lentil, in which *F*. *avenaceum* reduced seedling survival [11].

Under field conditions, inoculation with *F*. *avenaceum*, *F*. *proliferatum*, and *F*. *oxysporum* individually, or with a combination of *F*. *proliferatum* and *F*. *oxysporum*, resulted in significant yield reductions for both of the cultivars examined, highlighting the virulence of all three *Fusarium* spp. on canola. Similarly, previous studies also reported severe yield losses from Fusarium seedling blight and Fusarium wilt of canola [33]. These findings underscore the importance of managing Fusarium root rot to safeguard canola crops and ensure optimal yields. Nonetheless, the yield losses resulting from inoculation with *F*. *avenaceum* or *F*. *proliferatum* were more pronounced in 2022 than in 2021. In both years, seedling emergence was reduced when cultivars were inoculated with *F*. *proliferatum*, while the reductions caused by *F*. *avenaceum* were particularly prominent in 2022. These findings align with a study by Chang et al. [34], who reported similar losses in the stand establishment (22–31%) and seed yield (19%) of lupine caused by *F*. *avenaceum*. The greater yield losses observed in 2022 likely reflected more severe disease that year, even for similar treatments. While most *Fusarium* spp. are known to prefer warm temperatures [12,16], the very hot and dry conditions experienced in 2021 may not have been conducive to disease development. Moreover, the hot and dry weather in 2021 per se may have contributed to reduced seedling emergence, as suggested by the significant differences observed between the non-inoculated controls in 2021 and 2022; around 400 seedlings per plot were recorded in 2022, compared with fewer than 100 seedlings per plot in 2021. Previous studies have documented the influences of temperature and soil moisture on the severity of root rot caused by *Fusarium* spp. For example, Yan et al. [35] reported that *F*. *solani* and *F*. *tricinctum* caused the greatest root rot in soybean at 20 °C in sandy loam soil and at 15 °C in a silt loam. Additionally, they found that, when the temperature reached 28 °C, most infections occurred at soil moisture levels of 40% to 80% water holding capacity. Similarly, severe root rot symptoms were observed on lentil at temperatures ranging from 20 °C to 27.5 °C under controlled conditions, with fewer symptoms observed in warmer or cooler soils [36].

In the field experiments, co-inoculation with *F*. *proliferatum* and *F*. *oxysporum* had similar effects on the emergence and yield relative to inoculation with a single *Fusarium* species. In general, all inoculated treatments showed a reduction in these parameters with increasing disease severity. These findings align with previous studies investigating the effects of *F*. *avenaceum* on canola and faba bean [2,37], in which changes in growth parameters became more pronounced with increasing inoculum levels. Furthermore, in the current study, no significant differences were observed among treatments combining a high level of *F*. *proliferatum* (50 mL *Fp*: 0 mL *Fo*: 50 mL sand) and a high level of *F*. *oxysporum* (0 mL *Fp*: 50 mL *Fo*: 50 mL sand), a high level of *F*. *proliferatum* and a low level of *F*. *oxysporum* (50 mL *Fp*: 15 mL *Fo*: 35 mL sand), or a low level of *F*. *proliferatum* and a high level of *F*. *oxysporum* (15 mL *Fp*: 50 mL *Fo*: 35 mL sand). It is worth noting, however, that the treatment combining a moderate level of *F*. *proliferatum* and a moderate level of *F*. *oxysporum* (25 mL *Fp*: 25 mL *Fo*: 50 mL sand) resulted in a greater reduction in seedling emergence and yield than when a high level of a single *Fusarium* species was applied. Similar results were observed with respect to seedling emergence and root rot severity under greenhouse conditions. These findings suggest that there may be synergistic interactions between the two *Fusarium* species under certain combinations or inoculum concentrations. Further studies involving multiple strains of each species are needed to confirm the widespread occurrence of the synergistic phenomenon among different *Fusarium* species in canola root rot.

Synergistic interactions between pathogens can occur when they benefit mutually from biochemical signals essential for pathogenesis, or when they exchange resources necessary for survival, leading to functional complementation [38,39]. For instance, an additive interaction was reported between *F*. *oxysporum* and *R*. *solani* in causing root rot in soybean [40]. Likewise, it was observed that, when maize ears were inoculated with a spore mixture of *Fusarium graminearum* and *Fusarium verticillioides*, or when *F*. *graminearum* was sequentially followed by *F*. *verticillioides*, the competitiveness of the latter species improved [41]. Additionally, prior infection by *F*. *graminearum* was found to benefit subsequent infections by *F*. *verticillioides* [41]. Similar interactions have been observed in bacteria, where resources may be shared, benefiting all species while reducing mutual competition [42,43]. In contrast, competitive interactions were detected among four *Fusarium* spp. in causing Fusarium head blight of wheat [44]. These studies underscore the complexity of relations among pathogens and emphasize the need for further research to improve understanding of the mechanisms behind such interactions.

Co-inoculation with *F*. *proliferatum* and *F*. *oxysporum* under greenhouse conditions yielded results similar to those from the field experiments. In general, all inoculations led to a significant reduction in seedling emergence, seed weight per plant, and the number of pods per plant. Notably, disease severity at maturity was strongly and negatively correlated with both seed weight and pod number per plant, with regression analysis indicating a linear decline in these parameters as disease severity increased. At a maximum disease severity of 4.0, both yield parameters approached zero. These findings are similar to those reported by Wang et al. [45], who observed a decrease in seed yield and pod number per plant caused by blackleg disease in canola. By establishing a clear relationship between disease severity and yield parameters such as seed weight and pod production, the regression models developed in this study provide a quantitative understanding of the impact of Fusarium root rot on canola. 

In conclusion, all three *Fusarium* species were shown as capable of causing severe root rot in canola, reducing yields, as well as emergence and growth. Furthermore, co-inoculation with *F*. *proliferatum* and *F*. *oxysporum* resulted in greater disease severity than inoculation with each species on its own, suggesting possible synergistic interactions between these pathogens. The identification of significant negative linear correlations of seedling emergence, pod number per plant, and seed weight per plant with disease severity also underscores the need to implement more effective root rot management strategies. The knowledge from this study could be used to inform such strategies, and to more fully document the potential economic impact of Fusarium root rot in canola.

## 4. Materials and Methods

### 4.1. Isolates of Fusarium spp. and Inoculum Production

One isolate each of *F*. *proliferatum* (P002), *F*. *avenaceum* (F4A), and *F*. *oxysporum* (FOC4), originally collected from canola tissues showing symptoms of root rot [9], were included in this study. All isolates were stored at 4 °C on potato dextrose agar (PDA) until use. 

An inoculum of each isolate was generated separately on a wheat grain medium [46]. Briefly, grains of wheat (1 L) were soaked overnight in tap water, and then placed in a Hi Patch Mushroom Spawn Bag (Western Biologicals, Aldergrove, BC, Canada). The bag was sealed with a foam insert, secured with a collar, and autoclaved for 90 min. After it cooled to room temperature, the grain was inoculated with 0.5 cm diam. agar plugs excised from 14-day-old cultures of *F*. *proliferatum*, *F*. *avenaceum*, or *F*. *oxysporum* produced on PDA. The inoculated grains were mixed thoroughly by shaking and placed in an incubation chamber under darkness at room temperature for 5 weeks, allowing for complete colonization of the kernels. Subsequently, the inoculum was allowed to air-dry at 25 °C for 3 days, ground, and passed through a 2.0 mm mesh sieve. The grain inoculum was stored at 4 °C for a maximum of 2 months until further use.

### 4.2. Effects of F. proliferatum and F. avenaceum under Greenhouse Conditions

The abilities of *F*. *proliferatum* and *F*. *avenaceum* to cause root rot on canola, as well their impact on plant growth parameters, were evaluated under greenhouse conditions. The experiments were arranged in a randomized complete block design with five replicates, utilizing the canola hybrids ‘45CS40’, ‘L234’, ‘CS2000’, and ‘45H31’. In trials with *F*. *avenaceum*, the open-pollinated canola cultivar ‘Westar’ was included as an additional host. Each canola genotype was planted at a density of 10 seeds per cup in 473 mL cups (Uline, Toronto, ON, Canada) filled with 400 mL of Promix PGX potting medium (Sun-Gro Canada Inc., Seba Beach, AB, Canada). The grain inoculum of each fungal species was blended with the potting medium at specific ratios, determined based on a preliminary assessment of isolate aggressiveness. For *F*. *proliferatum*, the grain inoculum was mixed with the potting medium in the following ratios (*v*/*v*): 1:67, 1:100, 1:133, 1:200, 1:400, 1:1000, and 1:2000. These ratios resulted in inoculum densities ranging from 9 × 10^4^ to 3 × 10^3^ colony-forming units (cfu) per gram of potting mix. The grain inoculum of *F*. *avenaceum* was mixed with the potting mix (*v*/*v*) in the following ratios: 3:17, 3:22, 2:23, 3:47, 1:24, and 1:49. These ratios resulted in inoculum densities ranging from 9 × 10^5^ to 1.2 × 10^5^ cfu per gram of soil mixture. Controls did not receive grain inoculum from either species (i.e., 0:1 (*v*/*v*), 0%).

After inoculation, the cups were carefully transferred to a greenhouse and maintained at approximately 25 °C under a 12 h photoperiod. Seedling emergence was recorded on the 7th day after seeding. On the 21st day after seeding, the plants were gently uprooted and thoroughly washed with tap water to assess root rot severity, as described below. All experiments were repeated.

### 4.3. Effects of F. proliferatum and F. avenaceum under Field Conditions

The effects of *F. proliferatum* and *F. avenaceum* inoculation on the growth and yield of canola were assessed under field conditions. Two field experiments were conducted at the St. Albert Research Station (53°42′ N, 113°38′ W), St. Albert, Alberta, over two years (seeded on 4 June 2021 and 24 May 2022), with one site in 2021 and two sites in 2022. No occurrences of root rot disease were previously observed at the designated field site. Two widely cultivated canola hybrids, ‘45H31’ and ‘CS2000’, were sown in plots treated with grain inoculum, as previously described by Hwang et al. [3]. Each plot consisted of four 6 m rows with 30 cm spacing between rows, seeded at a rate of 0.7 g seeds per row. Trials were arranged in a randomized split-plot design with four replicates, with varieties as the main plot and different inoculum levels as subplots. For *F*. *avenaceum*, the grain inoculum was added with the canola seed at rates of 0 (control), 50, 100, and 150 mL per 6 m row. For *F*. *proliferatum*, grain inoculum was added with the canola seed at rates of 0 (control), 5, 10, and 15 mL per 6 m row. The relative rates of inoculum added for each species were based on a preliminary assessment of aggressiveness, as described above. Emergence at the seedling stage, disease severity at the flowering stage, and yield per plot at maturity were recorded for both experiments. 

### 4.4. Effects of F. proliferatum and F. oxysporum Alone or in Combination under Greenhouse Conditions

To assess the impacts of *F*. *proliferatum* and *F*. *oxysporum,* both individually and in combination, on root rot development and canola growth and yield, two repeated greenhouse experiments were conducted using the canola hybrids ‘45H31’ and ‘CS2000’. Canola seeds were sown in rectangular boxes (45 cm length × 30 cm width × 20 cm depth, 16 Qt.; Sterilite, Townsend, MA, USA), filled with 5 L of Promix PGX potting medium(Sun-Gro Canada Inc., Seba Beach, AB, Canada). The planting density was maintained at 12 seeds per row, with 3 rows per box.

Grain inocula of *F*. *proliferatum* and *F*. *oxysporum* were applied by mixing with the soilless mix in various ratios, determined based on a preliminary assessment. These ratios (*v*/*v*/*v*) included: 0 mL *F*. *proliferatum* (*Fp*): 0 mL *F*. *oxysporum* (*Fo*): 5000 mL soilless mix (control treatment), 75 mL *Fp*: 0 mL *Fo*: 5000 mL soilless mix, 0 mL *Fp*: 75 mL *Fo*: 5000 mL soilless mix, 22.5 mL *Fp*: 75 mL *Fo*: 5000 mL soilless mix, 75 mL *Fp*: 22.5 mL *Fo*: 5000 mL soilless mix, 75 mL *Fp*: 75 mL *Fo*: 5000 mL soilless mix, 37.5 mL *Fp*: 37.5 mL *Fo*: 5000 mL soilless mix, and 22.5 mL *Fp*: 22.5 mL *Fo*: 5000 mL soilless mix. Each treatment was replicated four times in separate boxes. The boxes were placed in a greenhouse at a temperature of approximately 25 °C under a 12 h photoperiod. The experiment was arranged in a randomized complete block design.

Seedling emergence was assessed on the 7th day after seeding. On the 21st day after seeding, plants from the second row were carefully uprooted and washed under tap water to assess root rot severity, as described below. The remaining two rows were allowed to grow until maturity, and disease severity, seed weight, and the number of pods were recorded for each plant at maturity. Seed weight and the number of pods per experimental unit (box) were calculated by adding the values obtained from all individual plants within the corresponding box.

### 4.5. Effects of F. proliferatum and F. oxysporum Alone or in Combination under Field Conditions

To evaluate the effects of *F*. *proliferatum* and *F*. *oxysporum* alone or in combination on canola growth and yield, another field experiment was conducted in 2022 at two sites at the St. Albert Research Station, where no prior instances of root rot disease had been identified. The canola hybrids ‘45H31’ and ‘CS2000’ were sown in plots treated with grain inoculum, as previously described by Hwang et al. [3]. Plots consisted of four 6 m rows with 30 cm spacing between rows, sown at a rate of 0.7 g canola seeds per row, as described above. Trials were arranged in a randomized split-plot design, with varieties as the main plot and inoculum levels as the subplots, and four replicates per treatment. The experiment was carried out with grain inocula of *F*. *proliferatum* and *F*. *oxysporum* mixed with sand at different ratios, which were then applied at a final rate of 100 mL per row. These ratios included: 0 mL *Fp*: 0 mL *Fo*: 100 mL sand (control treatment), 50 mL *Fp*: 0 mL *Fo*: 50 mL sand, 0 mL *Fp*: 50 mL *Fo*: 50 mL sand, 15 mL *Fp*: 50 mL *Fo*: 35 mL sand, 50 mL *Fp*: 15 mL *Fo*: 35 mL sand, 50 mL *Fp*: 50 mL *Fo*: 0 mL sand, 25 mL *Fp*: 25 mL *Fo*: 50 mL sand, and 15 mL *Fp*: 15 mL *Fo*: 70 mL sand. Seedling emergence, disease severity at flowering, and yield per plot at the maturity stage were recorded. 

### 4.6. Disease Ratings

Root rot severity was assessed on a 0–4 scale [11], for which: 0 = healthy roots; 1 = small, light-brown lesions on <25% of the tap root; 2 = brown lesions on 25–49% of the tap root; 3 = brown lesions on 50–74% of the tap root, tap root constricted; and 4 = tap root severely girdled, brown lesions on >75% of the tap root with limited lateral roots. In greenhouse studies, the final disease severity per experimental unit (cup or box) was calculated by averaging the values of all of the individual plants within each cup or box. In field experiments, the final root rot severity per plot was determined by averaging the values of 20 representative plants randomly selected from each plot. 

### 4.7. Seedling Emergence and Seed Weight

Seedling emergence was determined by counting all surviving plants per experimental unit (cup or box) in the greenhouse or per plot in the field. The seed weight was calculated by adding the yield of all individual plants within each experimental unit in the greenhouse or per plot in the field.

### 4.8. Weather Data Acquisition

Weather data (precipitation and temperature) for the St. Albert Research Station were collected using the Current and Historical Alberta Weather Station Data Viewer (https://acis.alberta.ca/acis/weather-data-viewer.jsp; accessed on 13 November 2022), Government of Alberta.

### 4.9. Data Analysis

All data sets were evaluated for homogeneity of variance with ANOVA, using R Studio [47]. Differences were considered significant at *p* ≤ 0.05 unless otherwise noted. If there was a significant interaction suggested between repetition (site or year) and treatment, the data were analyzed separately. Linear regression analysis was performed to evaluate the relationships between root rot severity and plant yield parameters under greenhouse conditions.

## Figures and Tables

**Table 1:** Comparison among different treatments on canola seedling emergence, root rot severity, and yield following inoculation with *Fusarium proliferatum* under field conditions in Edmonton, Alberta, in 2021 and 2022.

Cultivar	Treatment *^a^*	Emergence *^b^*		Disease Severity *^c^*		Yield (kg/ha) *^d^*		Yield Losses (%) *^e^*	
		2021	2022	2021	2022	2021	2022	2021	2022
‘45H31’	Control	96.8 A	426 a	0.25 A	0.323 a	2.30 A	3.92 a		
	Low	53.4 B	321 b	0.94 B	1.579 b	2.14 A	3.42 ab	6.96%	12.76%
	Moderate	29.9 C	337 b	1.41 C	1.979 c	2.13 A	3.41 ab	7.39%	13.01%
	High	18.0 C	286 b	1.72 D	2.078 c	2.07 A	2.91 b	10.00%	25.77%
‘CS2000’	Control	77.9 A	396 a	0.20 A	0.329 a	2.45 A	4.24 a		
	Low	51.2 B	331 ab	1.01 B	1.699 b	2.02 AB	3.47 b	17.55%	18.16%
	Moderate	25.3 C	311 bc	1.45 C	1.881 bc	2.12 AB	2.99 bc	13.47%	29.48%
	High	12.7 C	260 c	1.74 D	2.132 c	1.72 B	2.91 c	29.80%	31.37%

*^a^* Control refers to non-inoculated check plots; Low, Moderate, and High indicate treatments receiving low, moderate, and high inoculum levels, respectively. With regards to result columns, superscript letters indicate the following: *^b^* seedling emergence per plot; *^c^* average root rot disease severity (0–4 scale) per plot; *^d^* yield per hectare, calculated from the plot yield of each treatment; *^e^* yield losses, expressed as the percentage reduction for each inoculated treatment in relation to its corresponding control. Means in a column and category followed by the same uppercase or lowercase letter do not differ based on the LSD at *p* ≤ 0.05.

**Table 2:** Comparison among different treatments on canola seedling emergence, disease severity, and yield following inoculation with *Fusarium avenaceum* under field conditions in Edmonton, Alberta, in 2021 and 2022.

Cultivar	Treatment *^a^*	Emergence *^b^*		Disease Severity *^c^*		Yield (kg/ha) *^d^*		Yield Losses (%) *^e^*	
		2021	2022	2021	2022	2021	2022	2021	2022
‘45H31’	Control	64.9 A	406 a	0.17 A	0.328 a	1.88 A	4.13 a		
	Low	59.8 AB	222 b	1.14 B	1.451 b	1.71 A	3.26 b	9.04%	21.07%
	Moderate	58.8 B	147 c	1.21 B	1.788 c	1.56 A	3.21 bc	17.02%	22.28%
	High	57.6 B	123 c	1.29 B	2.095 d	1.51 A	2.74 c	19.68%	33.66%
‘CS2000’	Control	60.4 A	390 a	0.40 A	0.501 a	2.08 A	3.95 a		
	Low	59.7 A	240 b	1.07 B	1.580 b	1.93 A	3.06 b	7.21%	22.53%
	Moderate	56.2 A	180 bc	1.21 B	1.735 c	1.71 A	2.96 b	17.79%	25.06%
	High	55.9 A	131 c	0.93 B	2.015 d	1.71 A	2.75 b	17.79%	30.38%

*^a^* Control refers to non-inoculated check plots; Low, Moderate, and High indicate treatments receiving low, moderate, and high inoculum levels, respectively. With regards to result columns, superscript letters indicate the following: *^b^* seedling emergence per plot; *^c^* average root rot disease severity (0–4 scale) per plot; *^d^* yield per hectare, calculated from the plot yield of each treatment; *^e^* yield losses, expressed as the percentage reduction for each inoculated treatment in relation to its corresponding control. Means in a column and category followed by the same uppercase or lowercase letter do not differ based on the LSD at *p* ≤ 0.05.

**Table 3:** Comparison among different treatments on emergence and root rot severity in the canola cultivars ‘45H31’ and ‘CS2000’ following inoculation with *Fusarium proliferatum* and *Fusarium oxysporum*, alone or in combination, under greenhouse conditions.

Treatment *^a^*	‘45H31’			‘CS2000’		
	Emergence *^b^*	Disease Severity (Seedling Stage) *^c^*	Disease Severity (Maturity) *^d^*	Emergence *^b^*	Disease Severity (21 Days) *^c^*	Disease Severity (Maturity) *^d^*
Control	31.38 A	0.00 A	0.00 A	32.50 a	0.00 a	0.00 a
75 *Fp*: 0 *Fo*: 5000 mix	18.62 B	3.31 B	3.00 B	24.62 bc	2.83 bc	2.94 b
0 *Fp*: 75 *Fo*: 5000 mix	16.38 BC	3.01 B	3.17 BCD	27.25 ab	2.56 b	2.81 b
22.5 *Fp*: 75 *Fo*: 5000 mix	12.50 BC	3.15 B	3.29 BCDE	23.00 bc	2.86 bc	2.97 bc
75 *Fp*: 22.5 *Fo*: 5000 mix	9.25 CD	3.41 B	3.49 DE	20.88 bc	3.06 bc	2.98 bc
75 *Fp*: 75 *Fo*: 5000 mix	4.88 D	3.59 B	3.60 E	11.88 d	3.34 c	3.39 d
37.5 *Fp*: 37.5 *Fo*: 5000 mix	9.75 CD	3.47 B	3.35 CDE	17.38 cd	2.79 bc	3.30 cd
22.5 *Fp*: 22.5 *Fo*: 5000 mix	17.38 B	2.99 B	3.09 BC	25.62 ab	2.60 b	2.98 bc

*^a^* Control, no inoculum (0 mL *F. proliferatum* (*Fp*): 0 mL *F. oxysporum* (*Fo*): 5000 mL soilless mix); 75 *Fp*: 0 *Fo*: 5000 mix = 75 mL *Fp*: 0 mL *Fo*: 5000 mL soilless mix; 0 *Fp*: 75 *Fo*: 5000 mix = 0 mL *Fp*: 75 mL *Fo*: 5000 mL soilless mix; 22.5 *Fp*: 75 *Fo*: 5000 mix = 22.5 mL *Fp*: 75 mL *Fo*: 5000 mL soilless mix; 75 *Fp*: 22.5 *Fo*: 5000 mix = 75 mL *Fp*: 22.5 mL *Fo*: 5000 mL soilless mix; 75 *Fp*: 75 *Fo*: 5000 mix = 75 mL *Fp*: 75 mL *Fo*: 5000 mL soilless mix; 37.5 *Fp*: 37.5 *Fo*: 5000 mix = 37.5 mL *Fp*: 37.5 mL *Fo*: 5000 mL soilless mix; and 22.5 *Fp*: 22.5 *Fo*: 5000 mix = 27.5 mL *Fp*: 27.5 mL *Fo*: 5000 mL soilless mix. With regards to result columns, superscript letters indicate the following: *^b^* seedling emergence (number of plants) per box; *^c^* root rot disease severity (0–4 scale) at 21 days after seeding; *^d^* root rot disease severity (0–4 scale) at maturity. Means in a column and category followed by the same uppercase or lowercase letter do not differ based on the LSD at *p* ≤ 0.05.

**Table 4:** Comparison among different treatments on seed weight and pod number of the canola cultivars ‘45H31’ and ‘CS2000’ following inoculation with *Fusarium proliferatum* and *Fusarium oxysporum*, alone or in combination, at maturity under greenhouse conditions.

Treatment *^a^*	‘45H31’				‘CS2000’			
	Seed Weight (g) *^b^*		Pod Number *^c^*		Seed Weight (g) *^b^*		Pod Number *^c^*	
	1st	2nd	1st	2nd	1st	2nd	1st	2nd
Control	17.70 A	68.90 a	975 A	1855 a	12.71 A	64.00 a	645 A	2009 a
75 *Fp*: 0 *Fo*: 5000 mix	4.58 B	15.50 b	208 B	462 b	4.03 B	18.90 b	162 B	540 b
0 *Fp*: 75 *Fo*: 5000 mix	3.14 B	16.30 b	170 B	448 b	4.41 B	23.30 b	184 B	504 b
22.5 *Fp*: 75 *Fo*: 5000 mix	3.75 B	13.40 b	171 B	385 b	4.91 B	16.50 b	178 B	380 b
75 *Fp*: 22.5 *Fo*: 5000 mix	3.71 B	13.90 b	153 B	388 b	4.38 B	23.50 b	169 B	494 b
75 *Fp*: 75 *Fo*: 5000 mix	3.88 B	13.70 b	132 B	410 b	3.96 B	18.40 b	130 B	388 b
37.5 *Fp*: 37.5 *Fo*: 5000 mix	4.13 B	21.60 b	149 B	541 b	3.85 B	18.30 b	127 B	405 b
22.5 *Fp*: 22.5 *Fo*: 5000 mix	4.64 B	22.60 b	182 B	551 b	4.61 B	23.20 b	162 B	560 b

*^a^* Control, no inoculum (0 mL *F. proliferatum* (*Fp*): 0 mL *F. oxysporum* (*Fo*): 5000 mL soilless mix); 75 *Fp*: 0 *Fo*: 5000 mix = 75 mL *Fp*: 0 mL *Fo*: 5000 mL soilless mix; 0 *Fp*: 75 *Fo*: 5000 mix = 0 mL *Fp*: 75 mL *Fo*: 5000 mL soilless mix; 22.5 *Fp*: 75 *Fo*: 5000 mix = 22.5 mL *Fp*: 75 mL *Fo*: 5000 mL soilless mix; 75 *Fp*: 22.5 *Fo*: 5000 mix = 75 mL *Fp*: 22.5 mL *Fo*: 5000 mL soilless mix; 75 *Fp*: 75 *Fo*: 5000 mix = 75 mL *Fp*: 75 mL *Fo*: 5000 mL soilless mix; 37.5 *Fp*: 37.5 *Fo*: 5000 mix = 37.5 mL *Fp*: 37.5 mL *Fo*: 5000 mL soilless mix; and 22.5 *Fp*: 22.5 *Fo*: 5000 mix = 27.5 mL *Fp*: 27.5 mL *Fo*: 5000 mL soilless mix. With regards to result columns, superscript letters indicate the following: *^b^* total seed weight per box; *^c^* total pod number per box. 1st, first repeat; 2nd, second repeat of experiment. Means in a column and category followed by the same uppercase or lowercase letter do not differ based on the LSD at *p* ≤ 0.05.

**Table 5:** Comparison among different treatments on canola seedling emergence, disease severity, and yield of ‘45H31’ and ‘CS2000’ following inoculation with *Fusarium proliferatum* and *Fusarium oxysporum*, alone or in combination, under field conditions in Edmonton, Alberta, in 2022.

Treatment *^a^*	‘45H31’				‘CS2000’			
	Emergence *^b^*	Disease Severity (Flowering Stage) *^c^*	Yield (kg/ha) *^d^*	Yield Losses (%) *^e^*	Emergence *^b^*	Disease Severity (Flowering Stage) *^c^*	Yield (kg/ha) *^d^*	Yield Losses (%) *^e^*
Control	276.8 A	0.399 A	3.98 A		278.1 a	0.398 a	3.92 a	
50 *Fp*: 0 *Fo*: 50 sand	182.2 BC	1.751 B	3.28 B	17.59%	189.4 b	1.604 b	3.44 ab	12.24%
0 *Fp*: 50 *Fo*: 50 sand	162.0 BCD	1.728 B	3.22 B	19.10%	151.8 b	1.795 b	3.09 bcd	21.17%
15 *Fp*: 50 *Fo*: 35 sand	146.1 CD	1.748 B	3.25 B	18.34%	148.0 b	1.859 b	3.00 bcd	23.47%
50 *Fp*: 15 *Fo*: 35 sand	158.9 BCD	1.741 B	3.25 B	18.34%	160.0 b	1.741 b	3.34 bc	14.80%
50 *Fp*: 50 *Fo*: 0 sand	81.8 E	1.889 B	2.60 C	34.67%	67.9 c	1.764 b	2.72 e	30.61%
25 *Fp*: 25 *Fo*: 50 sand	128.6 D	1.581 B	3.21 B	19.35%	102.0 c	1.741 b	2.85 cd	27.30%
15 *Fp*: 15 *Fo*: 70 sand	193.6 B	1.684 B	3.57 AB	10.30%	164.0 b	1.718 b	3.23 bcd	17.60%

*^a^* Control, no inoculum (0 mL *F. proliferatum* (*Fp*): 0 mL *F. oxysporum* (*Fo*): 100 mL sand); 50 *Fp*: 0 *Fo*: 50 sand = 50 mL *Fp*: 0 mL *Fo*: 50 mL sand; 0 *Fp*: 50 *Fo*: 50 sand = 0 mL *Fp*: 50 mL *Fo*: 50 mL sand; 15 *Fp*: 50 *Fo*: 35 sand = 15 mL *Fp*: 50 mL *Fo*: 35 mL sand; 50 *Fp*: 15 *Fo*: 35 sand = 50 mL *Fp*: 15 mL *Fo*: 35 mL sand; 50 *Fp*: 50 *Fo*: 0 sand = 50 mL *Fp*: 50 mL *Fo*: 0 mL sand; 25 *Fp*: 25 *Fo*: 50 sand = 25 mL *Fp*: 25 mL *Fo*: 50 mL sand; and 15 *Fp*: 15 *Fo*: 70 sand = 15 mL *Fp*: 15 mL *Fo*: 70 mL sand. With regards to result columns, superscript letters indicate the following: *^b^* seedling emergence (number of plants) per plot; *^c^* root rot disease severity (0–4 scale) at the flowering stage; *^d^* yield per hectare, calculated from the plot yield for each treatment; *^e^* yield losses are the percentage reduction for each inoculated treatments in relation to corresponding control. Means in a column and category followed by the same uppercase or lowercase letter do not differ based on the LSD at *p* ≤ 0.05.

## Data Availability

The data presented in this study are available upon request from the corresponding author.

## Supplementary Materials

The following supporting information can be downloaded at: https://www.mdpi.com/article/10.3390/plants12173020/s1, Figure S1: Precipitations and mean temperatures at the site used in field trials, St. Albert, AB, 2021–2022; Table S1: Extreme high temperature (>30 °C) at the St. Albert research station in field trials, 2021–2022.
