# Supplementary material for: Characterization of the Virulence and Yield Impact of Fusarium Species on Canola (Brassica napus)
Source: Plants (Basel). 2023 Aug 22;12(17):3020. doi: 10.3390/plants12173020 (PMC10490129; doi:10.3390/plants12173020)
Supplement: Supplementary file 1 [file plants-12-03020-s001.zip › plants-2556934-supplementary.pdf]

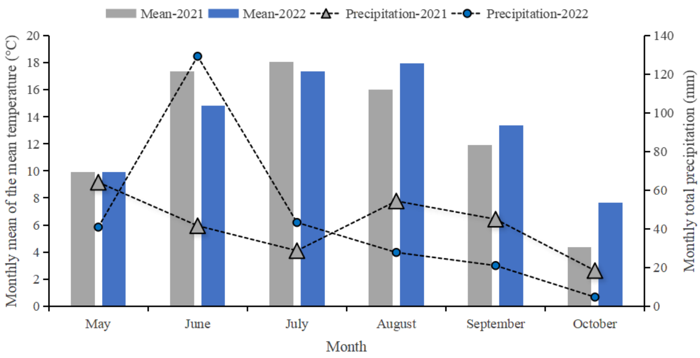

Figure S1. Precipitations and mean temperatures at the site used in field trials, St. Albert, AB, 2021-2022.

Table S1. Extreme high temperature (>30°C) at the St. Albert research station in field trials, 2021-2022

Date	Max Temp (°C)	Date	Max Temp (°C)
26-06-2021	30.8	28-07-2022	31.7
27-06-2021	30.7	13-08-2022	30.7
28-06-2021	35.2	19-08-2022	32.5
29-06-2021	35.2	20-08-2022	31
30-06-2021	35.9	26-08-2022	30.1
01-07-2021	34.3	30-08-2022	30.9
08-07-2021	31.4	31-08-2022	31.8
09-07-2021	32	02-09-2022	31.4
10-07-2021	32.5	03-09-2022	33.7
14-07-2021	30.8	04-09-2022	31.6
31-07-2021	30.1		
05-08-2021	31.1		
13-08-2021	32.2		
14-08-2021	32.3		
